# Carpet-dust chemicals as measures of exposure: Implications of variability

**DOI:** 10.1186/1742-7622-9-2

**Published:** 2012-03-23

**Authors:** Todd P Whitehead, John R Nuckols, Mary H Ward, Stephen M Rappaport

**Affiliations:** 1 Division of Environmental Health Sciences, University of California, Berkeley, CA, USA; 2 Department of Environmental and Radiological Health Sciences, Colorado State University, Fort Collins, CO, USA; 3 Division of Cancer Epidemiology and Genetics, National Cancer Institute, National Institutes of Health, Department of Health and Human Services, Bethesda, MD, USA

## Abstract

**Background:**

There is increasing interest in using chemicals measured in carpet dust as indicators of chemical exposures. However, investigators have rarely sampled dust repeatedly from the same households and therefore little is known about the variability of chemical levels that exist within and between households in dust samples.

**Results:**

We analyzed 9 polycyclic aromatic hydrocarbons, 6 polychlorinated biphenyls, and nicotine in 68 carpet-dust samples from 21 households in agricultural communities of Fresno County, California collected from 2003-2005. Chemical concentrations (ng per g dust) ranged from < 2-3,609 for 9 polycyclic aromatic hydrocarbons, from < 1-150 for 6 polychlorinated biphenyls, and from < 20-7,776 for nicotine. We used random-effects models to estimate variance components for concentrations of each of these carpet-dust chemicals and calculated the variance ratio, λ, defined as the ratio of the within-household variance component to the between-household variance component. Subsequently, we used the variance ratios calculated from our data, to illustrate the potential effect of measurement error on the attenuation of odds ratios in hypothetical case-control studies. We found that the median value of the estimated variance ratios was 0.33 (range: 0.13-0.72). Correspondingly, in case-control studies of associations between these carpet-dust chemicals and disease, given the collection of only one measurement per household and a hypothetical odds ratio of 1.5, we expect that the observed odds ratios would range from 1.27 to 1.43. Moreover, for each of the chemicals analyzed, the collection of three repeated dust samples would limit the expected magnitude of odds ratio attenuation to less than 20%.

**Conclusions:**

Our findings suggest that attenuation bias should be relatively modest when using these semi-volatile carpet-dust chemicals as exposure surrogates in epidemiologic studies.

## Background

Semi-volatile chemicals can accumulate in carpets over years and decades [1-3], and thus their concentrations in carpet dust could be useful surrogates for long-term indoor exposures in epidemiological studies [2,4-6]. Moreover, because dust ingestion or inhalation could be responsible for significant chemical exposures in young children [7-9], levels of chemicals in dust may be particularly relevant in studies of childhood diseases.

Although many researchers have measured chemicals in dust [10-12], few have sampled dust repeatedly in the same households [13-16] or characterized the variability of dust measurements within and between households [17,18]. In two studies that reported variance components of dust levels (of pesticides, lead, and phenanthrene), large variance ratios (*i.e*., ratio of within-household variance component to between-household variance component, designated here as λ) were observed [17,18]. Since, the degree of exposure measurement error increases directly with λ, large values of this ratio indicate imprecise exposure classification. In an epidemiological study, exposure misclassification will tend to result in the observation of risk estimates that are smaller than the true risks, a phenomenon referred to as attenuation bias. To employ carpet-dust concentrations as surrogates for chemical exposure with confidence, investigators first need to know how variable these measurements are within a given household, that is, they need some measure of their reliability.

Our objective in this analysis was to quantify the reliability of carpet-dust chemical concentrations as exposure measures for future epidemiological studies. We analyzed 9 polycyclic aromatic hydrocarbons (PAHs), 6 polychlorinated biphenyls (PCBs), and nicotine (as a surrogate for tobacco smoke) in repeated carpet-dust samples. These semi-volatile chemicals are particularly suitable for measurement in household dust because they persist in the indoor environment [10], and their long-term exposures have been associated with health effects [2,6,19,20]. Using random-effects models of repeated carpet-dust measurements, we estimated variance ratios for each of these chemicals. Subsequently, using our variance ratios, we estimated the amount of attenuation bias that would be expected to occur in independent case-control studies that used these carpet-dust chemicals as exposure measures.

## Methods

### Study households

We obtained dust samples from 21 households in Fresno County, California, from 2003-2005, as part of an investigation to estimate chemical exposures in residences located in agricultural communities. The study protocols were approved by the Institutional Review Boards at Colorado State University and the National Cancer Institute, and we obtained written informed consent from all participating subjects.

### Collection of carpet dust

We collected carpet-dust samples using a high-volume surface sampler (HVS3) as previously described [21]. Briefly, the interviewer selected a room on the side of the residence that faced agricultural crops, marked an approximately 4-foot by 6-foot area of a carpet or rug with tape, and vacuumed the surface in 3-inch strips, making four passes back and forth on each strip, until a 10 mL of fine dust had been collected. With few exceptions, all repeated samples we collected from a given household were from the same room. The median number of measurements per household was *n *= 3 (range of *n*: 1-7) and the median duration between repeated visits was 5 months (range of 3-15 months).

### Laboratory chemical analysis

We analyzed nine PAHs [benzo(*a*)anthracene, chrysene, benzo(*a*)pyrene, benzo(*b*)fluoranthene, benzo(*k*)fluoranthene, indeno(*1,2,3-c,d*)pyrene, dibenzo(*a,h*)anthracene, coronene, and dibenzo(*a,e*)pyrene], 6 PCBs (PCB 105, PCB 118, PCB 138, PCB 153, PCB 170, and PCB 180), and nicotine in dust samples as previously described [21]. Briefly, we sieved each dust sample using a 100-mesh stainless steel sieve (< 150 μm), extracted 0.5 g of fine dust with either a 1:1 hexane:acetone mixture (PAHs, PCBs) or methylene chloride (nicotine), then cleaned the extract using solid phase extraction (for PAHs and PCBs), and analyzed the concentrated eluate with gas chromatography-mass spectrometry (GC-MS) using p, p-dibromophenyl and d_12_-benzo(*e*)pyrene as internal standards for quantitation. PAHs and PCBs were analyzed using an RTx-5 MS column (30 M, 0.25 mm id, 0.25 μm film) with a GC oven temperature programmed from 130-220°C at 2°/min and then 220-330°C at 10°/min. Nicotine was analyzed using a DB-1701 column (30 M, 0.25 mm id, 0.15 μm film) with the GC oven temperature programmed 130-220°C at 2°C/min and then 220-280°C at 10°/min.

### Statistical analysis

Since the chemical concentrations were approximately log-normally distributed, we used the natural log-transformed values for all statistical analyses. We assigned all values below the limit of detection a concentration equal to the limit of detection divided by the square root of 2 [22]. We excluded chemicals that had detection rates less than 75% from the random-effects modeling (*i.e*., PCB 105, PCB 118, and PCB 170).

### Random-effects models

To estimate variance components, we used the one-way random-effects model,

(1) $$Y_{ij}=\text{ln }X_{ij}=\mu_{Y}+b_{i}+e_{ij},$$

for *i *= 1,2,...,*k *households and *j *= 1,2,...,*n *repeated measurements, where

*X_ij_*= the carpet-dust chemical concentration for the *i*^th ^household on the *j*^th ^repeated measurement;

*Y_ij_*= the natural log-transform of *X_ij_*;

*μ_Y_*= the true (logged) mean carpet-dust chemical concentration for the population;

*b_i_*= *μ_Yi _*-*μ_Y_*, and represents the random deviation of the *i*^th ^household's true mean (logged) carpet-dust chemical concentration, *μ_Yi_*, from *μ_Y_*;

*e_ij_*= *Y_ij _*- *μ_Yi_*, and represents the random deviation of the observed (logged) carpet-dust chemical concentration, *Y_ij_*, from *μ_Yi _*for the *i*^th ^household on the *j*^th ^repeated measurement.

We assumed *b_i _*and *e_ij _*are mutually independent and normally distributed random variables, with means of zero and variances $\sigma_{bY}^{2}$ and $\sigma_{wY}^{2}$, representing the between-household and within-household variances, respectively. These assumptions have been validated using repeated measurements of occupational chemical exposures [23-25].

Using Proc Mixed (SAS v.9.1, Cary, NC) we fit the model described in Equation 1 and estimated variance components (${\hat{\sigma }}_{bY}^{2}$,${\hat{\sigma }}_{wY}^{2}$, and ${\hat{\sigma }}_{Y}^{2}={\hat{\sigma }}_{wY}^{2}+{\hat{\sigma }}_{bY}^{2}$) and variance ratios, $\hat{\lambda }=\frac{{\hat{\sigma }}_{wY}^{2}}{{\hat{\sigma }}_{bY}^{2}}$. Subsequently, we estimated the expected concentration fold range for 95% of measurements (*i.e*., the expected ratio of the 97.5^th ^percentile concentration to the 2.5^th ^percentile concentration) from a single household [R^w0.95=exp(3.92×σ^wY)] and across all households in our study population [R^b0.95=exp(3.92×σ^bY)][25].

### Estimating attenuation bias

In the context of a case-control study, the following logistic model could be used to assess the risk of disease associated with a particular carpet-dust chemical:

(2) $$\text{Logit}\;Z_{i}=\text{ln}\left(\frac{Z_{i}}{Z_{i}-1}\right)=\beta_{0}+\beta_{1}\overset{-}{Y_{i}},$$

where

*Z_i_*= the disease status (1 or 0) of an individual in the i^th ^household and $\overset{-}{Y_{i}}$ = the (logged) mean carpet-dust chemical concentration for the i^th ^household.

In this case, the expected value of the estimated logistic regression coefficient, E[${\hat{\beta }}_{1}$], is related to the true logistic regression coefficient, *β_1_*, by the variance ratio, *λ*, as follows [26]:

(3) $$\text{E}\left[{\hat{\beta }}_{1}\right]=\frac{\beta_{1}}{1+\frac{\lambda }{n}}.$$

We define attenuation bias as the normalized difference between the expected value of the estimated logistic regression coefficient and the true logistic regression coefficient:

(4) $$B=\frac{\text{E}\left[{\hat{\beta }}_{1}\right]-\beta_{1}}{\beta_{1}}.$$

We used Equations 3 and 4 to estimate the amount of attenuation bias that would be expected in case-control studies using carpet-dust chemicals as independent variables in logistic regression analyses. For each chemical, using estimates of the variance ratio,$\hat{\lambda }$, from the application of the random-effects model (Equation 1), and an assumed true odds ratio of 1.5, we estimated the expected value for ${\hat{\beta }}_{1}$, the corresponding expected odds ratio, E[*OR*], and the expected amount of attenuation bias. It is worth noting that, in Equation 4, the magnitude of the attenuation bias is independent of the true odds ratio. In our calculations we assume that the variance ratio for the case and control populations are the same (*i.e*., measurement error is assumed to be non-differential).

Investigators can improve the precision of exposure estimates and, thereby, limit attenuation bias by making repeated exposure measurements and finding an average exposure level for each study subject over time. Combining Equations 3 and 4, it is possible to calculate the number of repeated measurements per household, *n*, that would be necessary to limit attenuation bias to a certain level as follows:

(5) $$n=\frac{\hat{\lambda }}{\frac{1}{1+B}-1}$$

Using our variance ratio estimates, we calculated the number of repeated measurements that would be necessary to limit the magnitude of attenuation bias to 20% in a case-control study using these carpet-dust chemicals as measures of exposure.

## Results

### Chemical concentrations in carpet dust

Our analyses included 21 households with 68 carpet-dust measurements. As shown in Table 1, individual chemical detection rates ranged from 38 to 100% and, as shown in Table 2, individual chemical concentrations ranged from less than the limit of detection to a maximum of 7,776 ng/g. We detected the 9 PAHs in a higher percentage of samples, and at higher median concentrations, than the 6 PCBs. The range in nicotine concentrations was larger than the range in concentrations of either PAHs or PCBs.

**Table 1:** Limits of detection and frequency of detection for 68 carpet-dust samples

Chemical	LOD, ng/g	Detected	% Detected
Nicotine^a^	20	44	77

Benzo(*a*)anthracene^b^	2	67	100

Chrysene	2	68	100

Benzo(*b*)fluoranthene	2	68	100

Benzo(*k*)fluoranthene	2	68	100

Benzo(*a*)pyrene	2	66	100

Indeno(*1,2,3-c,d*)pyrene	2	68	100

Dibenzo(*a,h*)anthracene	2	66	97

Coronene	4	68	100

Dibenzo(*a,e*)pyrene	4	68	100

PCB 105	1	26	38

PCB 118	1	45	66

PCB 138	1	52	76

PCB 153	1	59	87

PCB 170	2	28	41

PCB 180	2	51	75

Dust samples were collected from 21 households of Fresno County, California from 2003-2005

LOD = limit of detection

^a ^*N *= 57 for nicotine due to chemical interference during GC-MS analysis

^b ^*N *= 67 for benzo(*a*)anthracene due to chemical interference during GC-MS analysis

**Table 2:** Summary statistics for 68 carpet-dust samples

Chemical	Statistic	**Visit 1**, ***N*= 21**	**Visit 2**, ***N*= 15**	**Visit 3**, ***N*= 12**	**Visit 4**, ***N*= 9**	**Visit 5**, ***N*= 5**	**Visit 6**, ***N*= 4**	**Visit 7**, ***N*= 2**	**All Visits**, ***N*= 68**	Published Literature^a^
Nicotine	Minimum	< LOD	< LOD	< LOD	< LOD	< LOD	133	< LOD	< LOD	< 20

	Median	350	261	355	149	180	493	108	250	265

	Maximum	1544	7776	3964	1670	702	759	216	7776	35000

Benzo(*a*)anthracene	Minimum	7	11	5	8	11	8	8	5	< 2

	Median	28	28	33	21	15	59	27	27	25

	Maximum	1272	1014	1178	78	34	118	46	1272	834

Chrysene	Minimum	35	40	18	26	28	19	24	18	7

	Median	76	80	90	59	32	31	43	69	73

	Maximum	2867	2669	1887	186	105	111	62	2867	1547

Benzo(*b*)fluoranthene	Minimum	27	27	20	25	24	18	29	18	< 2

	Median	68	64	78	55	29	24	43	55	59

	Maximum	2660	2062	1874	173	99	34	58	2660	2450

Benzo(*k*)fluoranthene	Minimum	25	22	14	14	14	13	19	13	3

	Median	94	80	79	69	17	15	25	66	40

	Maximum	2413	938	1137	109	93	18	32	2413	814

Benzo(*a*)pyrene	Minimum	4	11	5	14	19	11	20	4	< 2

	Median	86	29	46	27	24	18	52	33	40

	Maximum	2127	1255	1980	164	38	31	83	2127	1948

Indeno(*1,2,3-c,d*)pyrene	Minimum	14	23	18	24	40	3	46	3	< 2

	Median	45	57	70	43	45	15	81	48	53

	Maximum	1988	1883	3609	294	92	65	116	3609	2371

Dibenzo(*a,h*)anthracene	Minimum	< LOD	6	4	4	4	4	6	< LOD	< 2

	Median	11	11	14	11	8	6	13	10	14

	Maximum	570	314	408	32	33	20	19	570	393

Coronene	Minimum	9	17	16	25	25	32	90	9	< 4

	Median	44	68	63	57	52	44	95	55	94

	Maximum	725	705	802	113	103	79	100	802	636

Dibenzo(*a,e*)pyrene	Minimum	6	10	9	12	16	29	71	6	< 4

	Median	16	20	24	17	33	46	82	21	27

	Maximum	491	375	1551	154	50	62	94	1551	713

PCB 105	Minimum	< LOD	< LOD	< LOD	< LOD	< LOD	< LOD	< LOD	< LOD	< 1

	Median	< LOD	< LOD	< LOD	1	< LOD	1	4	< LOD	< 1

	Maximum	54	13	10	11	10	6	8	54	49

PCB 118	Minimum	< LOD	< LOD	< LOD	< LOD	< LOD	< LOD	< LOD	< LOD	< 1

	Median	2	4	0.4	3	3	2	13	3	< 1

	Maximum	150	33	29	26	23	22	27	150	95

PCB 138	Minimum	< LOD	< LOD	< LOD	< LOD	3	< LOD	17	< LOD	< 1

	Median	2	6	3	4	7	13	21	5	< 1

	Maximum	118	46	31	28	25	23	26	118	145

PCB 153	Minimum	< LOD	< LOD	< LOD	< LOD	2	< LOD	16	< LOD	< 1

	Median	4	4	3	3	5	10	18	4	< 1

	Maximum	100	59	28	20	22	16	19	100	176

PCB 170	Minimum	< LOD	< LOD	< LOD	< LOD	< LOD	< LOD	< LOD	< LOD	< 2

	Median	< LOD	< LOD	< LOD	< LOD	3	3	6	< LOD	< 2

	Maximum	45	44	32	17	21	9	12	45	68

PCB 180	Minimum	< LOD	< LOD	< LOD	< LOD	2	< LOD	7	< LOD	< 2

	Median	4	3	2	2	4	6	25	3	< 2

	Maximum	114	97	60	37	39	30	43	114	108

Dust samples were collected from 21 households of Fresno County, California from 2003-2005

^a ^References for published literature summary statistics taken from Whitehead *et al. *[27] (nicotine); Whitehead *et al. *[3] (PAHs); and Ward *et al. *[2] (PCBs)

### Random-effects models

Table 3 shows the results of the analysis using random-effects models for the 13 chemicals with at least a 75% detection rate. For all models, the between-household variance component was greater than the within-household variance component (*i.e*., $\hat{\lambda }$< 1). The median within-household variance component estimate for PAHs was ${\hat{\sigma }}_{wY}^{2}$ = 0.38 (interquartile range, IQR: 0.21 - 0.42), for PCBs it was ${\hat{\sigma }}_{wY}^{2}$ = 0.41 (IQR: 0.36 - 0.51), and for nicotine it was ${\hat{\sigma }}_{wY}^{2}$ = 1.33. For each of the 13 individual chemicals, the within-household variance component ranged from ${\hat{\sigma }}_{wY}^{2}$ = 0.16 (coronene) to ${\hat{\sigma }}_{wY}^{2}$ = 1.33 (nicotine). Correspondingly, 95% of repeated coronene measurements from a household in our study population would be expected to lie within a 5-fold range versus a 92-fold range for repeated nicotine measurements. The median between-household variance component estimate for PAHs was ${\hat{\sigma }}_{bY}^{2}$ = 1.20 (IQR: 1.00 - 1.27), for PCBs it was ${\hat{\sigma }}_{bY}^{2}$ = 1.29 (IQR: 1.24 - 1.46), and for nicotine it was ${\hat{\sigma }}_{bY}^{2}$ = 1.85. For each of the 13 individual chemicals, the between-household variance component ranged from ${\hat{\sigma }}_{bY}^{2}$ = 0.77 [benzo(*k*)fluoranthene] to ${\hat{\sigma }}_{bY}^{2}$ = 1.85 (nicotine). Correspondingly, 95% of the mean benzo(*k*)fluoranthene concentrations from different households in our study population would be expected to lie within a 31-fold range versus a 207-fold range for mean nicotine levels.

**Table 3:** Variance parameter estimates from random-effects model regression analyses of repeated measurements of carpet-dust chemicals

Chemical	**Logged Study Mean **${\hat{\mu }}_{Y}$	**Total Variance **${\hat{\sigma }}_{Y}^{2}$	**Between-household variance **${\hat{\sigma }}_{bY}^{2}$	95% Confidence Interval	**Within-household variance **${\hat{\sigma }}_{wY}^{2}$	95% Confidence Interval	**Between-household fold range **${}_{b}{\hat{R}}_{0.95}$	**Within-household fold range **${}_{w}{\hat{R}}_{0.95}$	Variance Ratio $\hat{\lambda }$
Nicotine	5.5	3.18	1.85	(0.33, 3.37)	1.33	(0.75, 1.91)	207	92	0.72

Benzo(*a*)anthracene	3.5	1.55	1.24	(0.36, 2.11)	0.32	(0.19, 0.45)	78	9	0.26

Chrysene	4.5	1.28	1.07	(0.34, 1.80)	0.21	(0.12, 0.29)	58	6	0.19

Benzo(*b*)fluoranthene	4.4	1.36	1.20	(0.40, 1.99)	0.16	(0.10, 0.23)	73	5	0.13

Benzo(*k*)fluoranthene	4.3	1.27	0.77	(0.13, 1.41)	0.49	(0.29, 0.70)	31	16	0.64

Benzo(*a*)pyrene	3.8	2.07	1.41	(0.28, 2.55)	0.66	(0.38, 0.94)	106	24	0.47

Indeno(*1,2,3-c,d*)pyrene	4.2	1.69	1.27	(0.35, 2.20)	0.42	(0.25, 0.59)	83	13	0.33

Dibenzo(*a,h*)anthracene	2.6	1.70	1.30	(0.33, 2.27)	0.40	(0.23, 0.56)	88	12	0.31

Coronene	4.1	1.10	0.94	(0.30, 1.58)	0.16	(0.10, 0.23)	45	5	0.17

Dibenzo(*a,e*)pyrene	3.4	1.37	1.00	(0.25, 1.74)	0.38	(0.22, 0.53)	50	11	0.38

PCB 138	1.5	2.25	1.64	(0.38, 2.91)	0.60	(0.35, 0.85)	152	21	0.37

PCB 153	1.6	1.61	1.20	(0.31, 2.08)	0.41	(0.24, 0.58)	73	12	0.34

PCB 180	1.5	1.60	1.29	(0.39, 2.18)	0.32	(0.19, 0.44)	85	9	0.25

Dust samples were collected from 21 households of Fresno County, California from 2003-2005

### Attenuation bias estimates

Table 4 shows the amount of attenuation that would be expected in odds ratios if case-control studies were to use each of the carpet-dust chemicals as independent variables in logistic regression analyses. For each of the 13 chemicals with at least a 75% detection rate, expected bias was calculated using Equations 3 and 4 along with estimates of the variance ratio from Table 3. We found that, by definition, the magnitude of expected bias increased with the estimated variance ratio. For example, for the chemical with the smallest variance ratio [benzo(*b*)flouranthene, $\hat{\lambda }$ = 0.13], the expected odds ratio would be 1.43 assuming only one measurement from each household (*i.e*., *n *= 1), indicating a -12% bias (true odds ratio = 1.5). However, for the chemical with the highest variance ratio (nicotine, $\hat{\lambda }$ = 0.72); the expected odds ratio under the same conditions would be 1.27, a -42% bias.

**Table 4:** Attenuation bias due to measurement error

Chemical	**True Odds Ratio **(*OR*)	**True Logistic Regression Coefficient **(*β_1_*)	Estimated Variance Ratio $\hat{\lambda }$	**Expected Logistic Regression Coefficient **$\text{E}\left[{\hat{\beta }}_{1}\right]$	Expected Odds Ratio E[*OR*]	**Expected Attenuation Bias **(*B*)	**No. of Repeats to Limit Bias to 20% **(*n*)
Nicotine	1.50	0.41	0.72	0.24	1.27	-0.42	3

Benzo(*a*)anthracene	1.50	0.41	0.26	0.32	1.38	-0.21	2

Chrysene	1.50	0.41	0.19	0.34	1.40	-0.16	1

Benzo(*b*)fluoranthene	1.50	0.41	0.13	0.36	1.43	-0.12	1

Benzo(*k*)fluoranthene	1.50	0.41	0.64	0.25	1.28	-0.39	3

Benzo(*a*)pyrene	1.50	0.41	0.47	0.28	1.32	-0.32	2

Indeno(*1,2,3-c,d*)pyrene	1.50	0.41	0.33	0.30	1.36	-0.25	2

Dibenzo(*a,h*)anthracene	1.50	0.41	0.31	0.31	1.36	-0.23	2

Coronene	1.50	0.41	0.17	0.35	1.41	-0.15	1

Dibenzo(*a,e*)pyrene	1.50	0.41	0.38	0.29	1.34	-0.27	2

PCB 138	1.50	0.41	0.37	0.30	1.35	-0.27	2

PCB 153	1.50	0.41	0.34	0.30	1.35	-0.25	2

PCB 180	1.50	0.41	0.25	0.33	1.38	-0.20	1

Expected logistic regression coefficients were calculated using Equation 3 assuming a true odds ratio of 1.5, a case-control study without repeated measurements, and the variance ratios for carpet-dust chemicals measured in the 21 households of Fresno County, California from 2003-2005. Estimates of *B *and *n *were calculated using Equations 4 and 5, respectively

**Figure 1:**
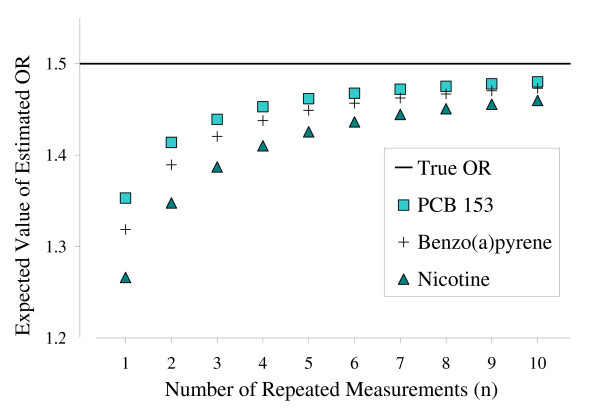
**Expected odds ratio attenuation**. Odds ratio attenuation in case-control studies that used (logged) carpet-dust chemical concentrations as measures of exposure, given various sampling strategies; for PCB 153 (squares), benzo(*a*)pyrene (crosses), and nicotine (triangles).

Figure 1 shows plots of the relationship between the expected odds ratio and the number of repeated measurements per household, using the estimated variance ratios from Table 3 and assuming a true odds ratio of 1.5 for PCB 153, benzo(*a*)pyrene, and nicotine. For each of the carpet-dust chemicals, Table 4 indicates that the number of repeated measurements necessary to limit attenuation bias to -20% ranged from 1 to 3 measurements per household.

## Discussion

Our results can guide epidemiologists in developing sampling strategies for using household dust as a medium for estimating exposures to PAHs, PCBs, or nicotine in their studies. Generally, investigators can improve the precision of their exposure estimates and limit attenuation bias by making repeated exposure measurements on each study subject. However, the analytical advantages of a repeated sampling design must be balanced with the practical concerns of a study's schedule and budget. By evaluating Equation 5 with our own variance ratio estimates, we provide future investigators a blueprint for obtaining precise exposure estimates without unnecessarily inflating study costs. As shown in Table 4, we found that, for each chemical we analyzed in carpet dust, three repeated dust measurements per household would be sufficient to reduce the magnitude of attenuation bias to less than 20%. From a practical standpoint, investigators could resample a particular carpet area as frequently as once a month. However, to observe (and adjust for) seasonal variation that may exist in carpet-dust chemical levels it would appropriate to sample over the course of an entire year. Moreover, for an investigator to estimate exposures that occurred in the distant past, it could be useful to collect samples over an even longer period of time. Indeed, for retrospective exposure assessment, increasing the duration of the dust collection period would enable an investigator to observe (and adjust for) any long-term time trends that may exist in carpet-dust chemical levels.

Moreover, if repeated sampling would not be feasible, Table 4 indicates that for 10 of the 13 chemicals analyzed, the expected magnitude of attenuation bias would still be less than 30%. Notably, nicotine, the most volatile chemical analyzed in our study, had a larger variance ratio than any of the PAHs or PCBs. Based on this observation, it is possible that carpet-dust concentrations of more volatile compounds will be more variable over time.

Our findings are based on a limited sample size (68 dust measurements from 21 households), and our variance ratio estimates are consequently somewhat imprecise (see Table 3). Moreover, our findings are based on dust measurements from only one surface type (carpets) and for only one general class of chemicals (semi-volatiles). However, we are confident that our findings will be externally valid and useful for other investigators measuring these same chemicals in dust. Notably, the dust concentrations of chemicals measured in our study (Table 2) were generally similar to the concentrations reported in recent studies of other households in California with respect to both the medians and the ranges of concentrations [2,3,27].

Unfortunately, it is difficult to compare our findings to those from two other studies that repeatedly sampled dust from the same households over time and reported corresponding variance components [17,18], because these studies published estimates for different chemicals in dust (*i.e*., pesticides, lead, and phenanthrene). However, our estimated variance ratios (Table 3) were quite similar to those we estimated using unpublished data from Egeghy *et al. *for several PAHs that were measured in household dust from both studies (Additional file 1). The similarity of variance ratios from two independent populations lends credibility to our findings and suggests that the levels of variability we observed in semi-volatile carpet-dust chemicals may be generalized to other populations.

In using the random-effects model to estimate variance components, we implicitly assume that each household has a true underlying mean dust concentration (for each chemical) that remains constant over the course of the study (*i.e*., *μ_Y_*+ *b_i_*). As such, we interpret any deviation from a household's true mean level as measurement error or random within-household variability. It is possible that some of the "random" variability that we observed is due to changes in the sources of chemical contamination in the homes, seasonal variations in temperature or ventilation practices, or other unaccounted-for factors that changed during the course of the study. Indeed, since our dust samples were collected over the period of 3 years, it is possible that true mean concentrations of chemicals in household dust did change somewhat over time. Consequently, the long-term timing of our sampling could have artificially inflated the within-household variance component, causing us to overestimate the variance ratios and the associated attenuation bias. Nevertheless, our random-effects model should provide a conservative estimate of the reliability of chemicals measured in carpet dust as measures of exposure.

One limitation of our method for predicting attenuation bias is that we specified that the variance ratios from the case and control populations were the same (*i.e*., measurement error was defined as non-differential) in Equation 3. In retrospective case-control studies, carpet-dust chemicals will be measured after disease diagnosis. In this scenario, case subjects could be more likely to change their behaviours between diagnosis and dust collection. If cases alter behaviours that result in changes to carpet-dust chemical levels, differential measurement error could occur. In the more complex situation in which case and control populations have differential measurement errors, Equation 3 would be only approximate. We were unable to evaluate whether variance ratios for concentrations of carpet-dust chemicals actually differ for case and control populations.

## Conclusions

In summary, we found that estimates of variance ratios of carpet-dust PAHs (0.13 ≤ $\hat{\lambda }$ ≤ 0.64), PCBs (0.25 ≤ $\hat{\lambda }$ ≤ 0.37), and nicotine ($\hat{\lambda }$ = 0.72) were modest for the 21 homes in our study area. Though based on a limited number of measurements (*N *= 68), our findings suggest that the use of carpet-dust samples as measures of exposure to these 13 chemicals will result in relatively small levels of attenuation bias due to exposure measurement error. Moreover, we have presented a simple guide for investigators to create efficient study designs that will limit bias in future studies that use dust to measure exposures to PAHs, PCBs, or nicotine.

## Competing interests

The authors declare that they have no competing interests.

## Authors' contributions

TPW performed statistical analyses (including the random effects modeling and the attenuation bias estimates) and drafted the manuscript. JRN participated in the design of the dust collection procedure, in sample collection, and in data processing. MHW participated in the design of the dust collection procedure, oversaw laboratory analyses, and participated in data processing. SMR supervised statistical analyses and provided critical feedback. All authors reviewed the initial manuscript, provided revisions, and approved the final manuscript.

## Supplementary Material

Additional file 1 **External variance parameter estimate comparison**. In this table we compare the variance parameter estimates from random-effects model regression analyses of repeated measurements of chemicals in dust collected from 2003-2005 in 21 households of Fresno County, California (Table 3) versus estimates for 50 households of Baltimore, Maryland sampled from 1995-1996 (based on unpublished data accompanying Egeghy *et al. *[18]). Click here for file
