# Supplementary material for: Carpet-dust chemicals as measures of exposure: Implications of variability
Source: Emerg Themes Epidemiol. 2012 Mar 23;9:2. doi: 10.1186/1742-7622-9-2 (PMC3368732; doi:10.1186/1742-7622-9-2)
Supplement: Additional file 1 — External variance parameter estimate comparison. In this table we compare the variance parameter estimates from random-effects model regression analyses of repeated measurements of chemicals in dust collected from 2003-2005 in 21 households of Fresno County, California (Table 3) versus estimates for 50 households of Baltimore, Maryland sampled from 1995-1996 (based on unpublished data accompanying Egeghy et al. [18]). [file 1742-7622-9-2-S1.DOC]

**Additional file 1 for ‘Carpet-dust chemicals as measures of exposure: Implications of variability’**

Todd P Whitehead, John R Nuckols, Mary H Ward, Stephen M Rappaport

**Variance ratio comparisons**

We used the model in Equation 1, to estimate variance ratios for unpublished data from Egeghy *et al.* [18], an independent study that repeatedly sampled dust from households over time. Egeghy *et al.* collected 126 house-dust samples from 50 households in Baltimore, MD from 1995-1996 using an HVS3 sampler. The authors reported variance components for only three chemicals in dust (chlorpyrifos, lead, and phenathrene), but made additional results available to the public (online at [www.epa.gov/heds](http://www.epa.gov/heds)). We calculated variance ratios for 7 PAHs that were analyzed in dust from both the Egeghy *et al.* study and our study [i.e. benzo(*a*)anthracene, chrysene, benzo(*b*)fluoranthene, benzo(*k*)fluoranthene, benzo(*a*)pyrene, indeno(*1,2,3-c,d*)pyrene, dibenzo(*a,h*)anthracene]. Additional file 1 compares the estimated variance components and variance ratios from the two studies*.* The median variance ratio from the data of Egeghy *et al.* was $\hat{\lambda}$ = 0.31 (interquartile range: 0.30 - 0.39) compared to our median of $\hat{\lambda}$ = 0.31 (interquartile range: 0.23 - 0.40) for concentrations of the 7 PAHs. The similarity of the variance ratios for PAHs measured in dust from two independent populations lends credibility to our findings and suggests that the levels of variability we observed may be generalized to other populations.

**Additional File 1 - External variance parameter estimate comparison**

|  | **Fresno County, CA** | | |  | **Baltimore, MD^a^** | | |
| --- | --- | --- | --- | --- | --- | --- | --- |
| **Chemical** | $\hat{\sigma}_{bY}^{2}$ | $\hat{\sigma}_{wY}^{2}$ | $\hat{\lambda}$ |  | $\hat{\sigma}_{bY}^{2}$ | $\hat{\sigma}_{wY}^{2}$ | $\hat{\lambda}$ |
| Benzo(*a*)anthracene | 1.24 | 0.32 | 0.26 |  | 1.54 | 0.48 | 0.31 |
| Chrysene | 1.07 | 0.21 | 0.19 |  | 1.51 | 0.43 | 0.29 |
| Benzo(*b*)fluoranthene | 1.20 | 0.16 | 0.13 |  | 1.46 | 0.59 | 0.40 |
| Benzo(*k*)fluoranthene | 0.77 | 0.49 | 0.64 |  | 1.86 | 0.55 | 0.29 |
| Benzo(*a*)pyrene | 1.41 | 0.66 | 0.47 |  | 1.92 | 0.57 | 0.30 |
| Indeno(*1,2,3-c,d*)pyrene | 1.27 | 0.42 | 0.33 |  | 1.96 | 0.74 | 0.38 |
| Dibenzo(*a,h*)anthracene | 1.30 | 0.40 | 0.31 |  | 1.18 | 0.54 | 0.45 |

^a^ Based on unpublished data accompanying Egeghy *et al.* [18]

**References**

1. Egeghy PP, Quackenboss JJ, Catlin S, Ryan PB. **Determinants of temporal variability in NHEXAS-Maryland environmental concentrations, exposures, and biomarkers**. *J Expo Anal Environ Epidemiol*. 2005 Sep; **15**(5):388-97.
